# The effect of oral anticoagulants on the incidence of dementia in patients with atrial fibrillation: A systematic review and meta-analysis

**DOI:** 10.1016/j.ijcrp.2024.200282

**Published:** 2024-05-07

**Authors:** Fakhar Latif, Muhammad Moiz Nasir, Komail K. Meer, Syed Husain Farhan, Huzaifa Ahmad Cheema, Adam Bilal Khan, Mohammad Umer, Wajeeh Ur Rehman, Adeel Ahmad, Muhammad Aslam Khan, Talal Almas, Sebastian Mactaggart, Abdulqadir J. Nashwan, Raheel Ahmed, Sourbha S. Dani

**Affiliations:** a Department of Internal Medicine, Dow University of Health Sciences, Karachi, Pakistan; b Department of Cardiology, King Edward Medical University, Lahore, Pakistan; c Department of Internal Medicine, United Health Services Hospital, Johnson City, NY, USA; d Department of Internal Medicine, Mass General Brigham - Salem Hospital, Salem, MA, USA; e Department of Internal Medicine, Guthrie Robert Packer Hospital, Sayre, PA, USA; f Department of Internal Medicine, University Hospitals Cleveland Medical Center, Cleveland, OH, USA; g Department of Medicine, Northumbria Hospitals NHS Foundation Trust, Newcastle upon Tyne, UK; h Hamad Medical Corporation, Doha, Qatar; i National Heart & Lung Institute, Imperial College London, London, UK; j Department of Cardiology, Royal Brompton Hospital, London, UK; k Department of Cardiology, Lahey Hospital and Medical Center, Burlington, MA, USA

**Keywords:** Anticoagulants, DOACs, Dementia

## Abstract

**Background:**

Dementia is a recognized complication of atrial fibrillation (AF). Oral anticoagulant (OAC) therapy can potentially be protective against this complication.

**Methods:**

A comprehensive search of MEDLINE and Embase for comparative observational studies reporting the efficacy of OAC therapy for the incidence of dementia in patients with AF was conducted from its inception until March 2023. Studies that had patients with prior use of OAC or with a previous history of dementia were excluded.

**Results:**

A total of 22 studies were included in this review involving 617,204 participants. The pooled analysis revealed that OAC therapy, including direct oral anticoagulants (DOACs) and vitamin K antagonists (VKAs), was associated with a reduced incidence of dementia in AF patients. Specifically, compared to non-OAC treatment, OACs demonstrated a significant reduction in dementia incidence (HR 0.68, 95 % CI [0.58, 0.80], p < 0.00001), with similar findings observed for DOACs (HR 0.69, 95 % CI [0.51, 0.94], p = 0.02) and VKAs (HR 0.73, 95 % CI [0.56, 0.95], p = 0.02). The comparison of DOAC vs VKA revealed that DOACs are associated with reduced risk of dementia (HR 0.87, 95 % CI [0.79, 0.96], p = 0.004).

**Conclusion:**

Our SR and meta-analysis showed that the use of OAC therapy is associated with a reduced risk of dementia in individuals with AF. However, our results are limited by the potential influence of confounding bias and significant heterogeneity in the analyses.

## 1 Introduction

Atrial fibrillation (AF) is a common type of cardiac rhythm disorder that is associated with increased mortality, morbidity and significant economic implications [1]. With the rise in AF-related hospitalization, it has become a major contributor to healthcare costs, resulting in an economic burden on the healthcare system [2]. Atrial fibrillation has been associated with a fivefold risk of stroke-a condition preceding dementia [3]. Additionally, several studies have claimed that atrial fibrillation can cause significant damage to the nervous tissue through silent cerebral infarcts, reduced cerebral blood flow, and chronic cerebral hypoperfusion, which subsequently results in cognitive decline [4,5]. Similarly, other studies indicated that a potential explanation for the relationship between dementia and atrial fibrillation could be their similar risk factors, such as sex, age, lifestyle habits, hypertension, diabetes, and heart disease [[6], [7], [8], [9], [10]].

These claims have led the researchers to believe that using oral anticoagulants (OAC) as the gold standard treatment for atrial fibrillation can also improve cognitive outcomes in patients [11,12]. Although this needs further investigation, it is believed that OACs could interrupt the blood coagulation cascade by inhibiting clotting factors, thereby preventing symptomatic or silent brain infarctions [12]. Furthermore, OACs improve overall brain health by attenuating neuro-inflammation by inhibiting protease-activated receptor-1 and 2 [13].

The available evidence on the effect of OACs on cognitive decline in atrial fibrillation patients is limited and insufficient to reach valid conclusions with far-reaching clinical implications. Therefore, our systematic review (SR) and meta-analysis aimed to investigate the efficacy of OACs on the incidence of dementia in patients with atrial fibrillation and determine which type of OAC treatment, vitamin K antagonists or direct oral anticoagulants, is more effective.

## 2 Methods

This SR and meta-analysis was conducted following the guidance presented in the Preferred Reporting Items for Systematic Reviews and Meta-Analyses (PRISMA) [14]. The protocol was registered with PROSPERO (CRD42023408750).

### 2.1 Inclusion and exclusion criteria

All observational studies and randomized control trials (RCTs) that included adult patients with AF to investigate the effect of OACs on the occurrence of dementia were considered eligible for the SR and meta-analysis. Studies that had patients with prior use of OACs or with a previous history of dementia were excluded. No particular language restriction was imposed. Any editorials, comments, reviews, and case reports were also excluded.

### 2.2 Data sources and search strategy

The databases used for the literature search were Embase (Elsevier; Amsterdam, Netherlands) and MEDLINE (PubMed interface), and the search was performed from inception until March 2023. ClinicalTrials.gov was also reviewed to find any published or unpublished trials on this topic; however, only the published trials were included in the final analysis. Reference lists of the articles in past related meta-analyses were also screened. The general search string used is as follows: (OAC OR Oral Anticoagulants OR VKA OR Vitamin K Antagonist OR Warfarin OR NOAC OR DOAC OR Direct Oral Anticoagulants OR Dabigatran OR Rivaroxaban OR Apixaban OR Edoxaban) AND (Dementia OR Vascular Dementia OR Alzheimer's Disease OR Lewy Body Dementia OR Cognitive Impairment OR Cognitive Decline) AND (Atrial Fibrillation OR AF OR Nonvalvular Atrial Fibrillation). The MeSH terms for each of the terms mentioned above were also used. Separate search strings for each database were created (Supplementary Table 1).

All the studies retrieved from each database were then exported to the EndNote reference management software, version 20.2.1 (Clarivate Analytics), where duplicates were identified and removed. The remaining articles were then carefully reviewed based on their titles and abstracts, followed by a full-text review to finalize the relevant studies. This was performed by two independent reviewers (F.L. and M.M.N.). Any dispute between these two authors on any study was resolved by a third reviewer (S.H.F).

### 2.3 Data extraction

The following data were extracted from the included studies: Study details (study year, first author name, country of origin, study design, follow-up period), patients’ characteristics (sex and average age), Mean CHA2DS2-VASc score. Moreover, our outcome of interest was the incidence of dementia, for which hazard ratios were extracted for the following comparisons: OAC vs. Non-OAC, DOAC vs. Non-OAC, VKA vs Non-VKA, and DOAC vs. VKA.

### 2.4 Quality assessment

The Newcastle-Ottawa quality assessment scale was used to evaluate the quality of each of the included observational studies based on three general parameters: Selection, Comparability, and Outcome [15]. Stars were awarded out of a maximum of 9 possible, and based on the total number of stars, studies were classified as being either “Poor quality,” “Fair quality,” or “Good quality.”

### 2.5 Statistical analysis

The statistical analysis of the pooled data was performed on Review Manager, version 5.4.1 (Copenhagen: The Nordic Cochrane Centre, The Cochrane Collaboration, 2014). A random-effects model was used considering the differences in the clinical setups where the studies were performed. The Higgins I^2^ statistic test was used to test for any potential heterogeneity in the studies, and a heterogeneity greater than 50 % was regarded as considerable [16]. To investigate the sources of heterogeneity, a leave-one sensitivity analysis was conducted where each study was excluded. Additionally, we stratified our analyses according to the type of study (prospective vs. retrospective), presence of observational window (with observational window vs. without observational window), and prior history of stroke. Publication bias was visually assessed through an inverted funnel plot using a fixed effects model. A 2-sided *P*-value ≤0.05 was considered statistically significant in all cases.

## 3 Results

### 3.1 Literature search

**Fig. 1:**
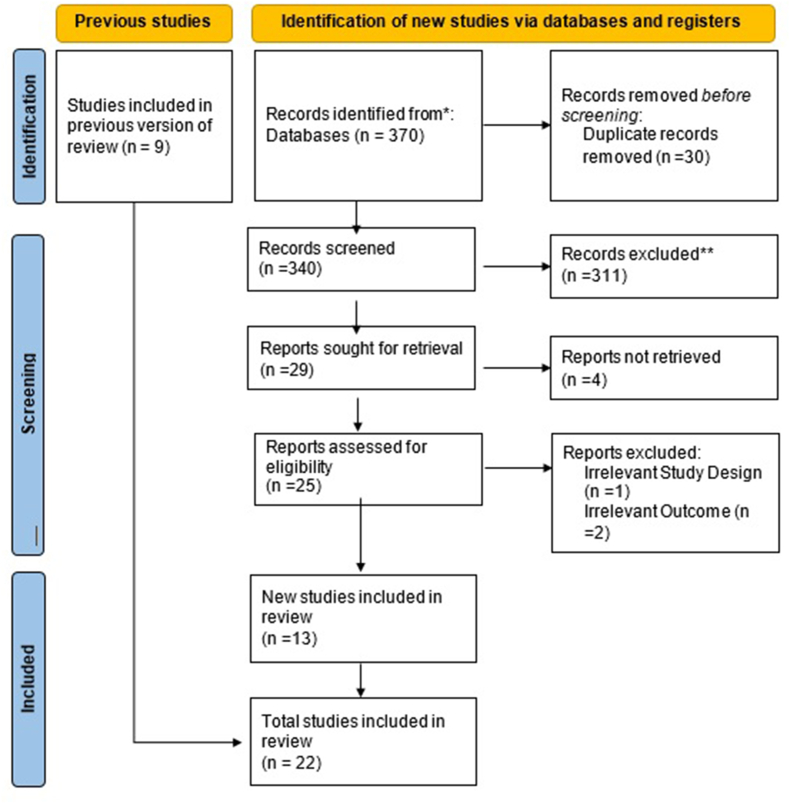
Study selection flow diagram presented according to the PRISMA statement.

Our extensive literature search identified 370 results **(PRISMA flowchart,** Fig. 1**).** After removing duplicates and irrelevant articles, twenty-two studies were pooled for the SR and meta-analysis [12,[17], [18], [19], [20], [21], [22], [23], [24], [25], [26], [27], [28], [29], [30], [31], [32], [33], [34], [35], [36], [37]].

### 3.2 General characteristics

All twenty-two articles included were observational studies, with two having a prospective design and the other twenty retrospectives. Almost all the studies were conducted in Europe, with a total number of 617,204 participants. The follow-up duration ranged from 243 days to 10 years. Meanwhile, nine studies specified the observational window, ranging from 14 days to 4 years. The summary of the baseline characteristics of the included studies is presented in Table 1. Almost all the included studies were of good quality with 9/9 scores, as indicated in Supplementary Table 2.

**Table 1:** Characteristics of included studies.

First author, year	Country	Study design	Patients, n	Age (Median [IQR] or Mean [SD])	Male, %	Follow-up time	AF detection	Dementia or CD definition	CHADS2 or CHA2DS2-VASc Score	Stroke Exclusion	Observational Window	OAC use defined as	Drugs of OACs	OACs use (%)	Comparison group	HR (95%CI)
Mongkhon P, 2020 [21]	United Kingdom	Retrospective cohort study	84521	73.0 (11.4)	54.63 %	5.9 years	Read codes for AF	Read codes for dementia/CI or the prescription of anti-dementia drugs	CHA2DS2-VASc Score 2.1 (1.3)	No	1 year	OACs within 60-days after AF diagnosis	Warfarin, dabigatran, rivaroxaban, apixaban, edoxaban	41.7 %	No treatment or antiplatelet use	0.90 (0.85–0.95)
Barber M, 2004 [18]	United Kingdom	Prospective cohort study	258	72 (66, 78)	46.00 %	3 years	NR	TICSm ≤20 and IQCODE score of 3.12/3.19	NR	No	No	OAC use at baseline and not altered during follow-up	Warfarin	64.0 %	No treatment or antiplatelet use	0.52 (0.26–1.07)
Ding M, 2018 [23]	Sweden	Prospective cohort study	470	80.9 (9.4), no including incidence AF	44 %, no including incidence AF	9 years	ECG, physician's diagnosis, ICD-10	DSM-IV criteria	NR	No	No	OAC use at baseline and during follow-up	NR	27.2 %	No treatment or antiplatelet use	0.40 (0.18–0.92)
Field T, 2019 [17]	United Kingdom	Retrospective cohort study	15276	70.1 (10.9)	61.20 %	25.7 months since observational period	ICD-10 and OPCS-4 codes	ICD-10 codes	CHA2DS2-VASc Score 3.2 (1.9)	No	2 years	Continuing OACs after a grace period of 30 days	Acenocoumarol, phenindione, warfarin, parenteral anticoagulants, apixaban, dabigatran, edoxaban, rivaroxaban	NR	No treatment or antiplatelet use	0.87 (0.70–1.08)
Friberg L, 2018 [12]	Sweden	Retrospective cohort study	444106	OAC users 73.7, non OAC users 75.7	OAC users 59.4 %, non OAC users 51.9 %	9 years	ICD-10	ICD-10	CHA2DS2-VASc score: OAC users = 3.43, nonOAC user = 3.49	No	No	Prescriptions filled up to 30 days after the first contact with AF during the inclusion period	Warfarin, phenprocuomon, NOACs	45.7 %	No treatment or antiplatelet use	0.71 (0.68–0.74)
Madhavan M, 2018 [24]	United States	Retrospective cohort study	2800	71.2 (14.6)	53.40 %	5 years (3.7)	ICD-9 or ECG	ICD-9	CHA2DS2-VASc score 3 (2–4)	No	6 months	Warfarin use within the first 90 days after AF diagnosis and had an INR ³1.5 at least one time	Warfarin	50.5 %	Non-OAC users	0.80 (0.64–0.99)
Nah M, 2020 [19]	Korea	Retrospective cohort study	34462	Prevalent AF 74.1 (6.7), incident AF 73.4 (6.3)	44.30 %	10 years	ICD-10	ICD-10	NR	No	4 years	Prescription of drugs that contained the main ingredient codes for warfarin and DOACs	Warfarin and DOACs	NR	No treatment or antiplatelet use	0.50 (0.47–0.52)
Krawczyk M, 2019 [20]	Canada	Retrospective cohort study	4596	Known AF 79.8 (7.4), inpatient AFDAS 79.7 (7.2), outpatient AFDAS 77.9 (6.5)	46.70 %	5.5 years (3.5)	ECG, ³24 h cardiac rhythm monitoring	From medical histories	Pre-stroke CHA2DS2-VASc score: known AF 3.7 (1.1), inpatient AFDAS 3.5 (1.1), outpatient AFDAS 3.5 (1.1)	All patients with first-ever ischemic stroke	No	From medical histories	Warfarin and DOACs	NR	No treatment or antiplatelet use	0.65 (0.58–0.72)
Marzona I, 2016 [22]	Italy	Retrospective cohort study	27431	78.40 (7.22)	47.48 %	10 years	ICD-9	ICD-9 or prescription for any anti-dementia drug	NR	No	No	From medical histories	OACs	36.4 %	No treatment or antiplatelet use	0.92 (0.83–1.01)
Bezabhe, 2022 [37]	Australia	Retrospective cohort study	18 813	71.9 ± 12.6 years,	52.90 %	3.7 ± 2.5 years	NR	NR	CHA2DS2-VASc score: OAC users = 2.9 ± 1.4 non-OAC users = 2.9 ± 1.5	Yes	No	From medical histories	Warfarin and DOACs	60.7 %	Non-OAC users	0.59 (0.44–0.80)
Wong C.K et al. [29]	Hong Kong	Retrospective cohort study	3284	76.4 ± 5.3 years,	51.60 %	3.6 years	From medical histories	From medical histories	CHA2DS2-VASc: 3.94 ± 1.44	Yes	14 days	From medical histories	Warfarin	18.7 %	No treatment or antiplatelet use	0.14 (0.05–0.39)
Hsu Y et al., 2021 [34]	Taiwan	Retrospective cohort study	12068	NR	NOAC: 59.5 % Warfarin: 59 %	NOAC: 3.27 years Warfarin: 3.08 years	ICD-9 & ICD-10	ICD-9 & ICD-10	CHA2DS2-VASc score: high stroke risk was defined as a score of ≥3 in women and a score of ≥2 in men; middle stroke risk was defined as a score of 2 in	No	90 days	OACs 90-days after AF diagnosis	Warfarin and DOACs	NR	No treatment	NR
Komatsu Y, 2022 [31]	Japan	Retrospective cohort study	17962	Non-OAC: 51.4 ± 11.7 OAC: 56.9 ± 9.5	Non-OAC: 63.4 % OAC: 82.3 %	OAC: 1.5 ± 1.3 Non-OAC: 2.2 ± 1.6	ICD-10	ICD-10	Women and a score of 1 in men; low stroke risk was defined as a score of 1 or 0 in women and a score of 0 in men.	No	1 year	Patients who did not have at least 6 months of follow-up after their AF diagnosis	Warfarin and DOACs	44.0 %	Non-OAC users	0.66 (0.40–1.09)
Sagris D., 2023 [27]	TriNetX (mainly US)	Retrospective cohort study	215 404	70.2 ± 12.1 years (DOAC: 70.3 ± 11.9 years VKA: 70 ± 12.3 years)	58.20 %	5 years and 10 years	ICD-10	ICD-10	NR	No	No	OAC therapy started within one month of atrial fibrillation or atrial flutter diagnosis	DOACs (Dabigatran, Apixaban, Rivaroxaban, Edoxaban) and VKA	100.00 %	VKA	1.01 (0.97, 1.05)
Rahman AA., 2023 [26]	United Kingdom	Retrospective cohort study	142 227	74.9 ± 10.0 years (OAC: 74.1 ± 9.2 years Non-OAC: 75.4 ± 10.5 years)	52.5 % (OAC: 56.3 % Non-OAC: 49.8 %)	Diagnosis of dementia, death, or end of study period (Dec 31, 2019)	Read codes for AF	Read codes for dementia	85.8 % CHA2DS2-VASc ≥2	No	6 months	OAC use in the first 3 months after cohort entry (index date for cohort - 1 Jan 1988)	DOACs (Dabigatran, Apixaban, Rivaroxaban) and Warfarin	41.64 %	Non-OAC users	0.87 (0.81, 0.93)
Grymonprez M., 2023 [25]	Belgium	Retrospective cohort study	237 012	NOAC: 75.7 ± 10.1 years VKA: 70.2 ± 12.0 years	NOAC: 53.4 % VKA: 53.9 %	NOAC: 1.5 ± 1.5 years VKA: 0.9 ± 1.4 years (OT analysis)	ICD-coded hospital discharge diagnosis	ICD-coded hospital discharge diagnosis	NOAC: 3.4 ± 1.7 VKA: 3.1 ± 1.9	No	No	OAC prescription during the study period 2013–2019	DOACs (Dabigatran, Apixaban, Rivaroxaban, Edoxaban) and VKA (Warfarin, Phenprocoumon, Acenocoumarol)	100.00 %	VKA	0.91 (0.85, 0.98)
Jacob V., 2021 [33]	USA	Retrospective cohort study	5254	72.4 ± 10.9 years	59.00 %	243 days	ICD-9 and 10	ICD-9 and 10	NR	No	No	OAC therapy initiated between June 2010 and December 2014 (inclusion criteria - atleast two INR measurements under CPAS supervision)	DOACs (Dabigatran, Apixaban, Rivaroxaban) and Warfarin	100.00 %	VKA/Warfarin	0.57 (0.16, 1.97)
Kim D., 2020 [11]	Korea	Retrospective cohort study	53 236	NOAC: 73 (66–78) years VKA: 70 (62–77) years	58.70 %	20.2 months	ICD-10	ICD-10	NOAC: 4 (3–6) VKA: 4 (3–6)	No	180 days	OAC initiation after AF diagnosis (therapy initiated between Jan 2013 and Dec 2016)	NOACS (Dabigatran, Apixaban, Rivaroxaban) and Warfarin	100.00 %	VKA/Warfarin	0.78 (0.68, 0.90)
Cadogan S.L., 2021 [36]	United Kingdom	Retrospective cohort study	39 200	76 (68–83) years	55.40 %	501 (199–978) days	ICD-10	Clinical read codes from primary care records	NR	No	No	OAC prescription following NVAF diagnosis between Jan 2012 and Dec 2018	DOACs (Dabigatran, Apixaban, Rivaroxaban) and VKAs (Warfarin, Phenprocoumon, Acenocoumarol)	100.00 %	VKA	0.84 (0.72, 0.98)
Chen N., 2018 [35]	USA	Retrospective cohort study	468 445	Dabigatran: 67 (13) Warfarin: 67 (13) Rivaroxaban: 67 (13) Warfarin: 68 (13) Apixaban: 69 (13) Warfarin: 69 (13)	DOAC: 62.60 % Warfarin: 62.20 %	0.7–2.2 years	ICD-9/health insurance claims data	ICD-9/claims data	Dabigatran: 3.1 (2.0) VKA: 3.0 (2.0) Rivaroxaban: 3.1 (1.9) VKA: 3.1 (1.9) Apixaban: 3.4 (2.0) VKA: 3.4 (1.9)	No	No	First OAC prescription atleast 90 days after enrollment (inclusion criteria for enrollment - one inpatient claim or two outpatient claims with AF diagnosis separated by atleast 7 days and <1 year)	DOACs (Dabigatran, Apixaban, Rivaroxaban) and Warfarin	100.00 %	VKA/Warfarin	0.79 (0.74, 0.84)
Lee S.R., 2021 [30]	South Korea	Retrospective cohort study	72 846	Warfarin: 70.1 ± 11.2 years Dabigatran: 71.7 ± 9.9 years Rivaroxaban: 72.9 ± 9.8 years Apixaban: 73.7 ± 9.9 years Edoxaban: 72.1 ± 10.0 years	DOAC: 56.50 % Warfarin: 59.50 %	1.3 ± 1.1 years	Claims data for AF (defined by ICD-10) Korean National Health Insurance System	Diagnosis codes or prescription for dementia	Warfarin: 3.8 ± 1.9 Dabigatran: 4.0 ± 1.7 Rivaroxaban: 4.0 ± 1.7 Apixaban: 4.3 ± 1.7 Edoxaban: 3.9 ± 1.6	No	No	AF patients who had ≥1 pharmacy claim for OAC between January 2014 and December 2017	DOACs (Dabigatran, Apixaban, Rivaroxaban, Edoxaban) and Warfarin	100.00 %	VKA/Warfarin	0.99 (0.93, 1.06)
Søgaard M., 2019 [28]	Denmark	Retrospective cohort study	33 617	60–69 years: 65.9 ± 2.7 years 70–79 years: 74.8 ± 2.9 years ≥80 years: 85.6 ± 4.1 years	60–69 years: 63.00 % 70–79 years: 54.3 % ≥ 80 years: 40.20 %	3.4 (SD 1.6) years	ICD-10	Hospital inpatient and outpatient clinical diagnosis of dementia recorded in National Patient Registry	60–69 years: 2.1 ± 1.2 70–79 years: 3.1 ± 1.3 ≥80 years: 4.0 ± 1.1	No	180 days	OAC prescribed after hospital AF diagnosis or <30 days before AF diagnosis	NOACs (Dabigatran, Apixaban, Rivaroxaban) and Warfarin	100.00 %	VKA/Warfarin	0.92 [0.48, 1.76]

AF = atrial fibrillation; CD = cognitive deficit; CHADS2 = congestive heart failure, hypertension, age ≥75 years, diabetes mellitus, stroke/transient ischemic attack; CHA2DS2-VASc = congestive heart failure, hypertension, age ≥75 years, diabetes mellitus, stroke/transient ischemic attack, vascular disease, age 65–74 years, sex category; TICSm = the modified 13-item version of the Telephone Interview for Cognitive Status; IQCODE = the Informant Questionnaire on Cognitive Decline in the Elderly; DSM-IV = Diagnostic and Statistical Manual of Mental Disorders (Fourth Edition); ICD-10 = International Classification of Diseases, Tenth Revision; MMSE = Mini-Mental State Examination; OPCS-4 = The Office of Population Censuses and Surveys-4; AFDAS = AF detected after stroke.

### 3.3 Quantitative analysis

#### 3.3.1 OAC versus non-OAC

The pooled analysis of 13 studies compared the effectiveness of OAC treatment in AF patients in reducing the incidence of dementia with the non-OAC group [12,[17], [18], [19], [20], [21], [22], [23], [24],^26^,^29^,^31^,^37^]. The analysis indicated that the use of OACs was associated with a reduced incidence of dementia (HR 0.70 [0.60, 0.81]; p < 0.00001; I^2^ = 95 %).

### 3.4 Subgroup analysis

**Fig. 2:**
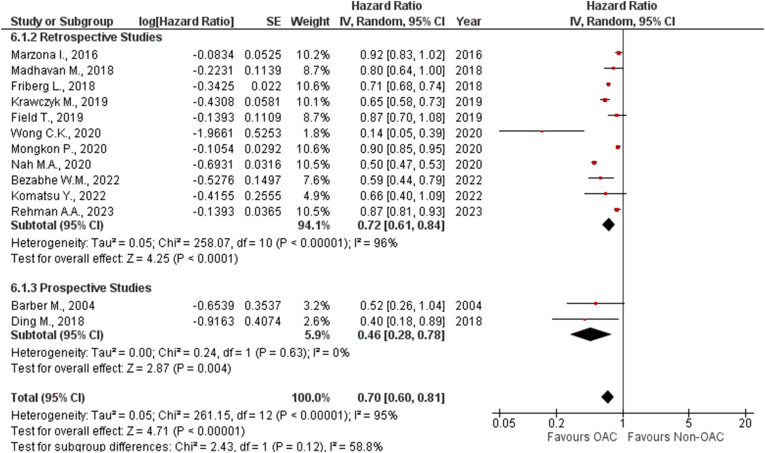
Forest plot of the pooled studies allocated to subgroups depending on study designs showing the comparison of OAC with non-OAC for risk of dementia.

***By Study Type:*** These studies were classified into the subgroups of prospective and retrospective cohorts. The subgroup analysis revealed that the retrospective studies reduced the risk of dementia (HR: 0.72 [0.61, 0.84]; P < 0.0001; I^2^ = 96 %), while the prospective studies lowered the risk of dementia by (HR: 0.46 [0.28, 0.78]; P = 0.004; I^2^ = 0 %) However, no potential differences between these study types were obtained (*P*_*interaction*_ = 0.12) (Fig. 2). There was considerable heterogeneity present (Tau^2^ = 0.05; Chi^2^ = 261.15, df = 12 (P < 0.00001); I^2^ = 95 %). The leave-one-out sensitivity analysis identified no study responsible for this high heterogeneity.

**Fig. 3:**
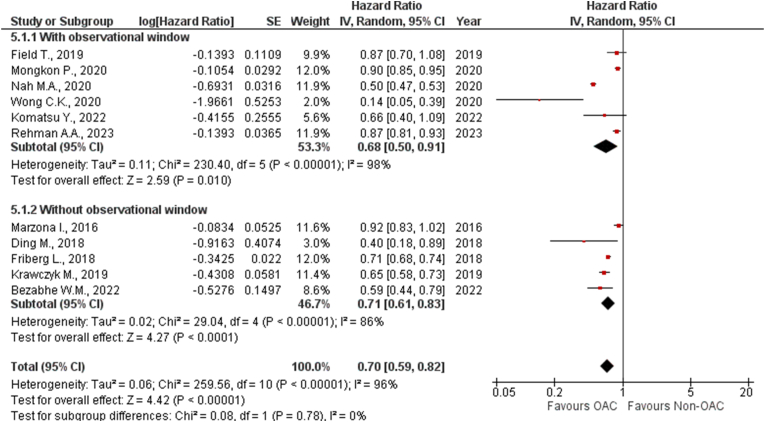
Forest plot of the pooled studies allocated to subgroups depending on observational window showing the comparison of OAC with non-OAC for risk of dementia.

***By Observational Studies:*** We subgrouped these studies based on those with and without any reported observational window. The analysis showed that studies with and without any reported observational windows significantly lowered the risk of dementia by 32 % (HR: 0.68 [0.50, 0.91]; P = 0.01; I^2^ = 98 %) and 29 % (HR: 0.71 [0.61, 0.83]; P < 0.0001; I^2^ = 86 %), respectively. A statistically significant association was observed between OAC use and reduced risk of dementia in both groups (*P*_*interaction*_ = 0.78) (Fig. 3).

**Fig. 4:**
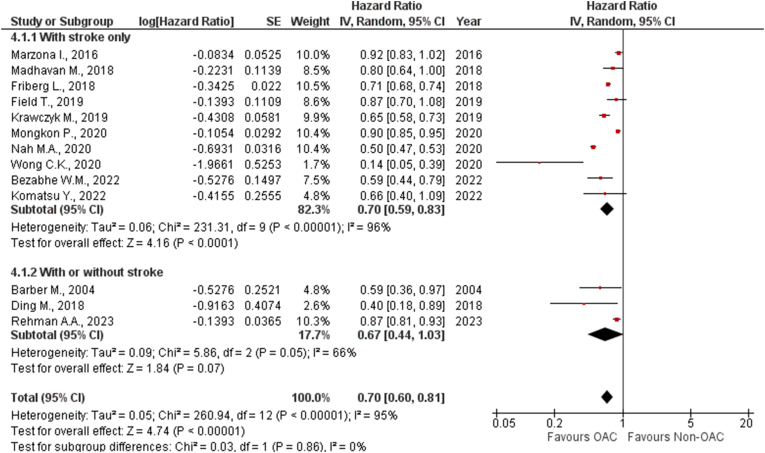
Forest plot of the pooled studies allocated to subgroups depending on prior history of stroke showing the comparison of OAC with non-OAC for risk of dementia.

***By Stroke History:*** We sub-grouped these studies into 2 groups: studies reporting patients with prior stroke history (HR: 0.70 [95 % CI: 0.59, 0.83]; P < 0.0001) and with or without stroke history (HR: 0.67 [95 % CI: 0.44, 1.03]; P = 0.07). No significant difference between these subgroups could be observed (*P*_*interaction*_ = 0.86) (Fig. 4). There was significant heterogeneity present in patients with prior stroke history (Tau^2^ = 0.06; Chi^2^ = 231.31, df = 9 (P < 0.00001); I^2^ = 96 %), while no heterogeneity was observed in studies with or without stroke history (Tau^2^ = 0.09; Chi^2^ = 5.86, df = 2 (P = 0.05); I^2^ = 66 %).

#### 3.4.1 Publication bias

The funnel plot of the included studies was asymmetrical, indicating a possible publication bias (Supplementary Fig. 1).

### 3.5 DOAC versus non-OAC

Five studies were pooled to compare the efficacy of DOAC with non-OAC to examine the incidence of dementia [12,21,31,34,37]. The remaining 17 studies were not included in this comparison due to the absence of reported data for DOACs. The SR and meta-analysis showed that the use of DOACs was significantly associated with a reduction in the incidence of dementia compared to non-OAC treatment (HR 0.69 95 CI [0.51, 0.94]; p = 0.02). There was considerable heterogeneity present (I^2^ = 91 %). In the sensitivity analysis, removing Friberg L. et al. reduced the heterogeneity to 65 %, which could be attributed to relatively longer follow-up duration than other included studies. (Supplementary Figs. 2 and 3).

### 3.6 VKA versus non-OAC

AF patients on VKAs (e.g., warfarin) were contrasted with patients on non-OAC treatment in 7 studies [12,21,24,29,31,34,37]. The other 15 studies included in the study did not report data for the comparison of VKAs with Non-OAC. The analysis exhibited that VKAs were associated with a more significant reduction in dementia incidence (HR 0.73 95 CI [0.56, 0.95]; p = 0.02) with considerable heterogeneity (I^2^ = 97 %). In the sensitivity analyses, Friberg L. et al. was identified as a cause of substantial heterogeneity, which could potentially be due to relatively longer follow-up duration of the study. (Supplementary Figs. 4 and 5).

### 3.7 DOAC versus VKA

Ten studies were pooled to compare the efficacy of DOACs with VKAs to examine the incidence of dementia [21,25,27,28,30,32,33,[35], [36], [37]]. The remaining 12 studies did not report data on this comparison. The pooled analysis revealed that DOACs significantly reduced the occurrence of dementia in atrial fibrillation patients by 13 % (HR 0.87 [95 % CI 0.79, 0.96]; p = 0.004) with significant heterogeneity (I^2^ = 86 %). The sensitivity analysis revealed that removal of Chen N., 2018 moderately reduced the heterogeneity (Supplementary Figs. 6 and 7).

## 4 Discussion

Our SR and meta-analysis showed that the use of OACs is associated with a reduced risk of dementia in AF patients. The subgroup analyses were consistent with this conclusion across the type of OAC used (DOACs or VKAs). However, our analyses is limited by high heterogeneity and suspicion of publication bias.

The results of this updated SR and meta-analysis are consistent with previous meta-analyses, which suggested that the use of OACs was more favorable in reducing the risk of dementia in atrial fibrillation patients [[38], [39], [40]] [[38], [39], [40]] [[38], [39], [40]]. Our SR and meta-analysis adds newer studies to the pooled analysis to provide more reliable and precise estimates than previous systematic reviews.

As the incidence of atrial fibrillation is expected to gradually rise worldwide, with some estimates suggesting a 150 % increase in the next four decades, the associated risk of dementia and cognitive impairment remains a looming threat to public health [41]. Although several models have been proposed to find an underlying mechanism behind this association, the direct relationship is likely multifactorial. Some of these factors include old age, vascular comorbidities, family history, and prior transient ischemic attacks. It is believed that the elevated risk factor for stroke post-atrial fibrillation complications (four to five-fold increased risk) may have the most significant causal relationship with dementia and cognitive decline [4]. Neuroimaging reveals silent brain infarcts may cumulate over an asymptomatic period, especially in patients with a history of cardiovascular diseases, invasive cardiac procedures, and congenital abnormalities, affecting the frontal lobes, white matter, and medial temporal lobes and manifesting as declining active brain functions [42,43]. Kalantarian et al., in their SR and meta-analysis, established a significant association between cognitive decline and stroke in populations with or without any history of stroke [44]. These findings were similar to those in our analysis, which demonstrated a statistically significant association between dementia and patients with or without a history of stroke. OACs, owing to their antithrombotic effects, may present as a potential mitigative measure for patients at risk of dementia. However, the elevated risk of dementia in patients without any prior stroke history poses questions about the hypothesis surrounding aggregating micro-infarcts following stroke events in patients with atrial fibrillation. The Rotterdam scan study was among the first few studies to discover a relation between atrial fibrillation and dementia, independent of stroke (OR 2.3; 95 % CI [1.4, 3.7]) [45]. Several underlying mechanisms have been proposed for these observations, such as decreased cerebral perfusion, atrial fibrillation-induced vascular inflammation, atrial fibrillation-associated shrinkage of the entorhinal cortex, and genetic factors (e.g., PITX2 locus), [5,[46], [47], [48]]. The inclusion of an observational window was aimed at trying to avoid overestimating the protective effects of OAC, where the pooled HR of studies with observational windows was assumed to be close to the real-time impact of OACs on dementia patients. It is noteworthy that the results for DOAC (HR 0.69 95 CI [0.51, 0.94]; p = 0.02) were slightly more favorable than VKA (HR 0.73 95 CI [0.56, 0.95]; p = 0.02).

Although the currently available guidelines suggest the use of OAC's in patients with AF and dementia according to their respective CHA_2_DS_2_‐VASc score, the differentiation between the subtypes of dementia and the outcomes of OAC administration remain undocumented. A study comparing the use of OACs between patients with Alzheimer's disease (AD) and vascular dementia in patients with AF, found and increased risk of ischemic stroke, re-hospitalization, and mortality in patients with vascular dementia, while patients with AD had a higher risk of non-traumatic ICH [49]. While the importance of distinguishing outcomes of OAC use in patients with AF and different types of dementia remain evident, the lack of available studies sets ground for future exploratory resource allocation.

The current SR and meta-analysis has some limitations. First, only a few studies reported dementia incidents in patients without any history of stroke. Second, most outcomes analyzed had high heterogeneity despite leave-one-out sensitivity analysis. Thirdly, since our SR and meta-analysis was based on data from observational studies, our findings are susceptible to confounding bias. Lastly, since our SR and meta-analysis was based on data from observational studies, our findings are susceptible to confounding bias. Although we used adjusted estimates from studies, wherever possible, residual bias could not be mitigated entirely. Our findings should be viewed as hypothesis-generating, and future RCTs should investigate the effect of OACs on the risk of dementia in atrial fibrillation patients.

## 5 Conclusion

Our SR and meta-analysis showed that the use of OAC therapy is associated with a reduced risk of dementia in individuals with atrial fibrillation. However, our results are limited by the potential influence of confounding bias and significant heterogeneity in the analyses. There is a need for randomized controlled studies to provide high-quality evidence on the association between the use of OAC and incident dementia in atrial fibrillation patients.

## Conflicts of interest

None to declare.

## Disclosure of funding

None to declare.

## CRediT authorship contribution statement

**Fakhar Latif:** Writing – original draft, Visualization, Validation, Software, Methodology, Investigation, Formal analysis, Data curation, Conceptualization. **Muhammad Moiz Nasir:** Writing – original draft, Visualization, Validation, Software, Methodology, Investigation, Formal analysis, Data curation, Conceptualization. **Komail K. Meer:** Writing – original draft, Methodology, Investigation, Data curation. **Syed Husain Farhan:** Writing – original draft, Methodology, Formal analysis, Data curation. **Huzaifa Ahmad Cheema:** Writing – review & editing, Writing – original draft, Visualization, Validation, Supervision, Resources, Project administration, Methodology, Investigation, Formal analysis, Data curation, Conceptualization. **Adam Bilal Khan:** Writing – original draft, Visualization, Validation, Investigation. **Mohammad Umer:** Writing – original draft, Visualization, Validation, Resources, Methodology, Investigation. **Wajeeh Ur Rehman:** Writing – original draft, Visualization, Validation, Methodology, Investigation, Formal analysis. **Adeel Ahmad:** Writing – review & editing, Methodology, Investigation, Formal analysis, Data curation. **Muhammad Aslam Khan:** Writing – review & editing, Visualization, Validation, Data curation. **Talal Almas:** Writing – review & editing, Supervision, Resources. **Sebastian Mactaggart:** Writing – review & editing, Visualization, Validation, Supervision, Resources, Methodology. **Abdulqadir J. Nashwan:** Writing – review & editing, Visualization, Validation, Supervision, Resources, Investigation. **Raheel Ahmed:** Writing – review & editing, Visualization, Validation, Supervision, Resources, Project administration, Methodology, Investigation. **Sourbha S. Dani:** Writing – review & editing, Visualization, Validation, Supervision, Methodology.
