# Supplementary material for: The effect of oral anticoagulants on the incidence of dementia in patients with atrial fibrillation: A systematic review and meta-analysis
Source: Int J Cardiol Cardiovasc Risk Prev. 2024 May 7;21:200282. doi: 10.1016/j.ijcrp.2024.200282 (PMC11101875; doi:10.1016/j.ijcrp.2024.200282)
Supplement: Multimedia component 1 [file mmc1.docx]

**Supplementary Materials:** Effect of Oral Anticoagulants on Incidence of Dementia in Patients with Atrial Fibrillation: A Systemic Review and Meta-Analysis

**Supplementary Figures**

**Supplementary Figure 1 Funnel plot of the 13 studies included in the meta-analysis**

**Supplementary Figure 2 Forest plot of the pooled studies showing the comparison of DOAC with Non-OAC for risk of dementia**

**Supplementary Figure 3 Forest plot of the pooled studies showing the sensitivity analysis for the comparison of DOAC vs. Non-OAC for risk of dementia**

**Supplementary Figure 4 Forest plot of the pooled studies showing the comparison of VKA with Non-OAC for risk of dementia**

**Supplementary Figure 5 Forest plot of the pooled studies showing the sensitivity analysis for the comparison of VKA vs. Non-OAC for risk of dementia**

**Supplementary Figure 6 Forest plot of the pooled studies showing the comparison of DOAC vs. VKA for risk of dementia**

**Supplementary Figure 7 Forest plot of the pooled studies showing the sensitivity analysis for the comparison of DOAC vs. VKA for risk of dementia**

**Supplementary Table**

**Supplementary Table 1: Search Strategies for the Retrieval of Included Studies**

**Supplementary Table 2: The Newcastle-Ottawa Scale for assessing the risk of bias of the included studies**


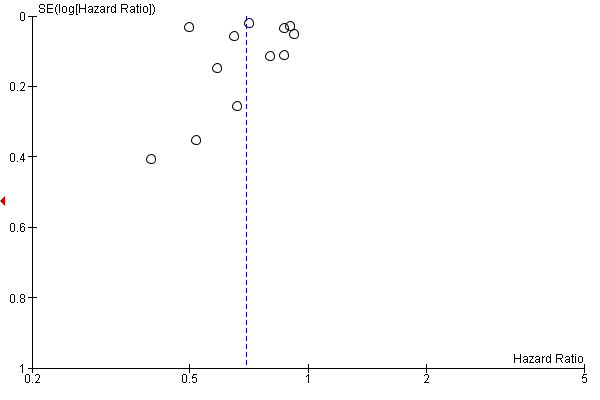


**Supplementary Figure 1 Funnel plot of the 13 studies included in the meta-analysis**


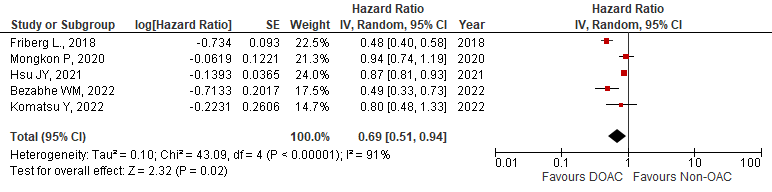


**Supplementary Figure 2 Forest plot of the pooled studies showing the comparison of DOAC with Non-OAC for risk of dementia**


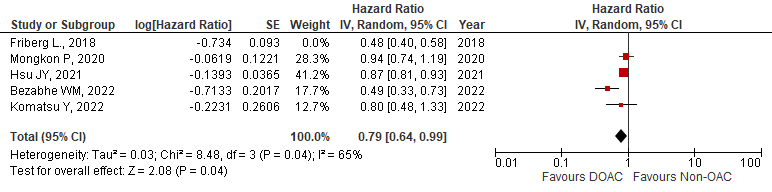


**Supplementary Figure 3 Forest plot of the pooled studies showing the sensitivity analysis for the comparison of DOAC vs. Non-OAC for risk of dementia**


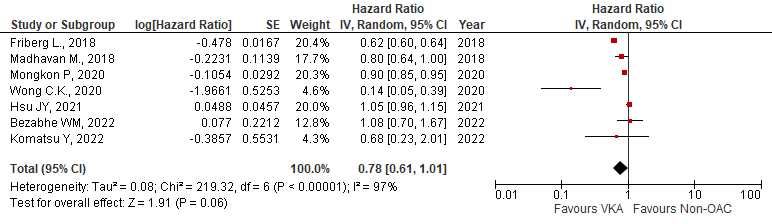


**Supplementary Figure 4 Forest plot of the pooled studies showing the comparison of VKA with Non-OAC for risk of dementia**


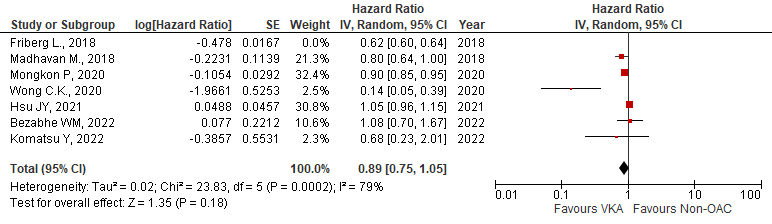


**Supplementary Figure 5 Forest plot of the pooled studies showing the sensitivity analysis for the comparison of VKA vs. Non-OAC for risk of dementia**


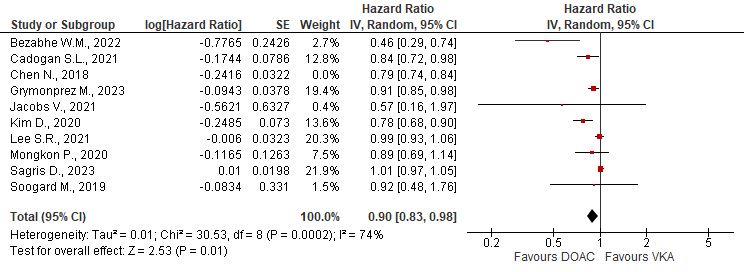

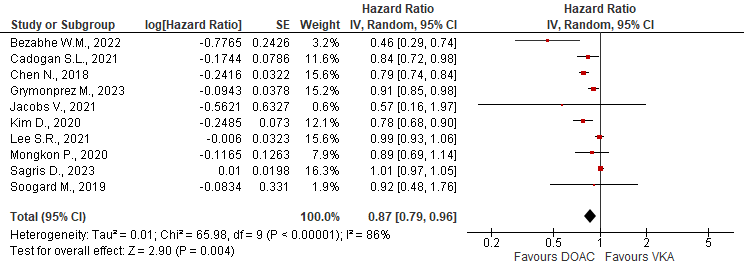


**Supplementary Figure 7 Forest plot of the pooled studies showing the sensitivity analysis for the comparison of DOAC vs. VKA for risk of dementia**

**Supplementary Figure 6 Forest plot of the pooled studies showing the comparison of DOAC vs. VKA for risk of dementia**

| Search number | Query | Results |
| --- | --- | --- |
|  | (Cognitive Decline) AND (Cognitive Decline[Title/Abstract]) | 31,797 |
|  | (Cognitive Impairment) AND (Cognitive Impairment[Title/Abstract]) | 80,301 |
|  | (Alzheimer's disease) AND (Alzheimer's disease[Title/Abstract]) | 151,573 |
|  | (Vascular Dementia) AND (Vascular Dementia[Title/Abstract]) | 7,862 |
|  | (Frontotemporal Dementia[Title/Abstract]) | 8,650 |
|  | (Lewy Body Dementia) AND (Lewy Body Dementia[Title/Abstract]) | 1,232 |
|  | (Mixed Dementia) AND (Mixed Dementia[Title/Abstract]) | 713 |
|  | dementia[Title/Abstract] | 138,504 |
|  | #1 OR #2 OR #3 OR #4 OR #5 OR #6 OR #7 OR #8 | 304,457 |
|  | atrial fibrillation[Title/Abstract] | 90,416 |
|  | AF[Title/Abstract] | 53,330 |
|  | A-fib[Title/Abstract] | 246 |
|  | Afib[Title/Abstract] | 518 |
|  | #11 OR #12 OR #13 OR #14 | 108,882 |
|  | anticoagulation[Title/Abstract] | 52,207 |
|  | oral anticoagulant[Title/Abstract] | 7,808 |
|  | oral anticoagulants[Title/Abstract] | 13,812 |
|  | OAC[Title/Abstract] | 7,392 |
|  | OACs[Title/Abstract] | 740 |
|  | vitamin K antagonist[Title/Abstract] | 3,642 |
|  | vitamin K antagonists[Title/Abstract] | 5,148 |
|  | VKA[Title/Abstract] | 2,480 |
|  | VKAs[Title/Abstract] | 1,438 |
|  | warfarin[Title/Abstract] | 27,436 |
|  | coumarin[Title/Abstract] | 13,350 |
|  | coumadin[Title/Abstract] | 1,124 |
|  | novel oral anticoagulant[Title/Abstract] | 387 |
|  | novel oral anticoagulants[Title/Abstract] | 1,189 |
|  | non-vitamin K antagonist oral anticoagulant[Title/Abstract] | 297 |
|  | non-vitamin K antagonist oral anticoagulants[Title/Abstract] | 1,191 |
|  | nonvitamin K antagonist oral anticoagulant[Title/Abstract] | 33 |
|  | nonvitamin K antagonist oral anticoagulants[Title/Abstract] | 120 |
|  | NOAC[Title/Abstract] | 1,850 |
|  | NOACs[Title/Abstract] | 2,225 |
|  | direct oral anticoagulant[Title/Abstract] | 1,639 |
|  | direct oral anticoagulants[Title/Abstract] | 4,898 |
|  | DOAC[Title/Abstract] | 2,733 |
|  | DOACs[Title/Abstract] | 3,238 |
|  | rivaroxaban[Title/Abstract] | 7,240 |
|  | dabigatran[Title/Abstract] | 5,869 |
|  | apixaban[Title/Abstract] | 4,864 |
|  | edoxaban[Title/Abstract] | 1,992 |
|  | #15 OR #16 OR #17 OR #18 OR #19 OR #20 OR #21 OR #22 OR #23 OR #24 OR #25 OR #26 OR #27 OR #28 OR #29 OR #30 OR #31 OR #32 OR #33 OR #34 OR #35 OR #36 OR #37 OR #38 OR #39 OR #40 OR #41 OR #42 | 103,394 |
|  | #9 AND #14 AND #43 | 370 |
|  |  |  |

**Supplementary Table 1 Search strategy used to retrieve relevant articles from databases**

| First author, year | Selection | | | | Comparability | | Outcome | | | Total NOS score |
| --- | --- | --- | --- | --- | --- | --- | --- | --- | --- | --- |
|  |  |  |  |  |  |  |  |  |  |  |
| Barber M, 2004 | ★ | ★ | ★ | ★ | ★ | ★ | ★ | ★ | ★ | 9/9 |
| Marzona I, 2016 | ★ | ★ | ★ | ★ | ★ | ★ | ★ | ★ | ★ | 9/9 |
| Friberg L, 2018 | ★ | ★ | ★ | ★ | ★ | ★ | ★ | ★ | ★ | 9/9 |
| Madhavan M, 2018 | ★ | ★ | ★ | ★ | ★ | ★ | ★ | ★ | ★ | 9/9 |
| Ding M, 2018 | ★ | ★ | ★ | ★ | ★ | ★ | ★ | ★ | ★ | 9/9 |
| Field T, 2019 | ★ | ★ | ★ | ★ | ★ | ★ | ★ | ★ | ★ | 9/9 |
| Krawczyk M, 2019 | ★ | ★ | ★ | ★ | ★ | ★ | ★ | ★ | ★ | 9/9 |
| Mongkhon P, 2020 | ★ | ★ | ★ | ★ | ★ | ★ | ★ | ★ | ★ | 9/9 |
| Nah M, 2020 | ★ | ★ | ★ | ★ | ★ | ★ | ★ | ★ | ★ | 9/9 |
| Hsu J, 2021 | ★ | ★ | ★ | ★ | ★ | ★ | ★ | ★ | ★ | 9/9 |
| Bezabhe W, 2022 | ★ | ★ | ★ | ★ | ★ | ★ |  | ★ | ★ | 8/9 |
| Komatsu Y, 2022 | ★ | ★ | ★ | ★ | ★ | ★ | ★ | ★ | ★ | 9/9 |
| Wong C, 2022 | ★ | ★ | ★ | ★ | ★ | ★ | ★ | ★ | ★ | 9/9 |
| Sagris D., 2023 | ★ | ★ | ★ | ★ | ★ | ★ | ★ | ★ | ★ | 9/9 |
| Rahman AA., 2023 | ★ | ★ | ★ | ★ | ★ | ★ | ★ | ★ | ★ | 9/9 |
| Grymonprez M., 2023 | ★ | ★ | ★ | ★ | ★ | ★ | ★ | ★ | ★ | 9/9 |
| Jacob V., 2021 | ★ | ★ | ★ | ★ | ★ | ★ | ★ | ★ | ★ | 9/9 |
| Kim D., 2020 | ★ | ★ | ★ | ★ | ★ | ★ | ★ | ★ | ★ | 9/9 |
| Cadogan S.L., 2021 | ★ | ★ | ★ | ★ | ★ | ★ | ★ | ★ | ★ | 9/9 |
| Chen N., 2018 | ★ | ★ | ★ | ★ | ★ | ★ | ★ | ★ | ★ | 9/9 |
| Lee S.R., 2021 | ★ | ★ | ★ | ★ | ★ | ★ | ★ | ★ | ★ | 9/9 |
| Søgaard M., 2019 | ★ | ★ | ★ | ★ | ★ | ★ | ★ | ★ | ★ | 9/9 |

**Supplementary Table 2 The Newcastle-Ottawa Scale for assessing the risk of bias of the included studies**
